# IgG:FcγRIIb Signaling on Mast Cells Blocks Allergic Airway Inflammation

**DOI:** 10.3390/ijms26146779

**Published:** 2025-07-15

**Authors:** Cynthia Kanagaratham, Yasmeen S. El Ansari, Kameryn N. Furiness, Hans C. Oettgen

**Affiliations:** 1 Department of Pediatrics, Boston Children’s Hospital, Boston, MA 02115, USA; cynthia.kanagaratham@childrens.harvard.edu (C.K.); yasmeen.elansari@chldrens.harvard.edu (Y.S.E.A.); kameryn.furiness@childrens.harvard.edu (K.N.F.); 2 Department of Pediatrics, Harvard Medical School, Boston, MA 02115, USA

**Keywords:** allergic lung inflammation, IgE, FcγRIIb

## Abstract

IgG antibodies, signaling via the inhibitory receptor, FcγRIIb, are potent inhibitors of IgE-mediated mast cell activation. We have previously reported that in addition to blocking mast cell degranulation, inhibitory IgG signals shut down a proinflammatory transcriptional program in which mast cells produce cytokines and chemokines known to drive type 2 tissue inflammation. To determine whether such effects of allergen-specific IgG can modulate allergic inflammation in vivo, we examined the airways of mice sensitized to ovalbumin (OVA) by intraperitoneal injection and then challenged with intranasal OVA. Pretreatment with allergen-specific IgG significantly reduced the recruitment of inflammatory cells, including macrophages and eosinophils, into the lungs of OVA-sensitized mice. The bronchoalveolar lavage fluid of OVA-challenged mice contained elevated levels of chemokine ligands (CCL2 and CCL24) and interleukin-5, a response that was markedly blunted in animals receiving allergen-specific IgG. IgG-treated animals exhibited attenuated allergen-induced production of IgE, IL-4, and IL-13, along with impaired OVA-induced goblet cell hyperplasia and *Muc5ac* expression and suppressed airway hyperresponsiveness, consistent with a shift away from a Th2 response. Using mice with a lineage-specific deletion of FcγRIIb, we demonstrated that each of these protective effects of IgG was dependent upon the expression of this receptor on mast cells. Overall, our findings establish that allergen-specific IgG can reduce allergen-driven airway inflammation and airway hyperresponsiveness and point to a mechanistic basis for the therapeutic benefit of aeroallergen-specific IgG therapy.

## 1. Introduction

Mast cells are innate immune cells armed for adaptive recognition of allergens by IgE antibodies tightly bound to their high-affinity receptor, FcεRI. Mast cells are expanded in the airways of allergic asthmatic patients and have been implicated in various aspects of asthma pathogenesis [1,2,3,4,5]. FcεRI crosslinking by allergens triggers signaling events that lead to mast cell degranulation. This process results in the immediate release of preformed vasoactive molecules from mast cell granules in addition to the rapid synthesis of eicosanoids [6,7]. Together, these mediators not only drive acute responses to inhaled aeroallergens but they also initiate a more gradual process of inflammation, promoting the recruitment of eosinophils and T cells, tissue remodeling, and airway obstruction [8,9]. Mice deficient in mast cell-derived mediators or treated with mast cell stabilizers exhibit reduced airway allergen-driven inflammation and hyperresponsiveness [3,10]. Mast cells additionally are a key tissue source of a variety of cytokines and chemokines that have been implicated in the pathogenesis of type 2 inflammation [11].

One of the disease-modifying treatment options for allergic disorders including asthma is immunotherapy, in which the administration of increasing doses of allergen leads to a gradual decrease in immunological sensitivity. One very consistent hallmark (as well as a biomarker of clinical efficacy) of immunotherapy to aeroallergens, venoms, and foods is a strong induction of allergen-specific IgG antibodies [12,13,14]. Similarly, naturally occurring IgG antibodies present in some allergen-exposed subjects producing specific IgE antibodies but not clinically allergic have been associated with protection from symptoms in a phenotype that some have referred to as “benign Th2 immunity” [15,16,17]. Recently, the passive administration of aeroallergen-specific IgG antibodies has been shown to impair mast cell activation and have clinical benefit in subjects with allergic rhinitis or asthma [18,19,20].

While IgG antibodies produced in response to immunotherapy or induced upon natural exposure or administered as therapeutics have often been referred to as “blocking” antibodies, implying steric masking of allergen epitopes from mast cell-bound IgE antibodies, their action is now known to be exerted via two distinct and potentially complementary mechanisms, (1) epitope masking and (2) the engagement of the inhibitory IgG Fc receptor, FcγRIIb, which suppresses the generation of phosphoprotein signaling intermediates generated upon FcεRI activation. We have found that inhibition through FcγRIIb signaling is more potent, requiring lower concentrations of IgG than epitope masking [21]. Studies of the effects of binding anti-IgE antibodies and their Fab fragments, or of CD23 to IgE, revealing the allosteric inhibition of IgE:FcεRI binding, suggest another potential mechanism for inhibition [22,23,24]. It is possible that the cross-linking of FcγRIIb and FcεRI might similarly exert allosteric effects on IgE binding.

In addition to inhibiting rapid mediator release, the delivery of inhibitory signals by IgG:FcγRIIb specifically shuts down a proinflammatory program of type 2 cytokine and chemokine gene expression that occurs in mast cells following IgE:allergen-mediated activation and blocks the recruitment of inflammatory cells to the peritoneal cavity following i.p. allergen injection in sensitized mice [21]. The relevance of this effect in emerging active immune responses is supported by studies in a food allergy model in which prophylactic treatment with allergen-specific IgG during sensitization diminishes the induction of type 2 allergic phenotypes, including IgE production and Th2 skewing [25]. This protective effect of IgG depends on the expression of FcγRIIb, as it is not evident in FcγRIIb knockout animals [25].

While observations of IgG induction in aeroallergen immunotherapy and the presence of IgG in subjects exhibiting the “benign Th2” phenotype suggest that IgG may exert its protective effects in aeroallergen-sensitive subjects, this possibility has not been directly tested. Here we use a mouse model to show that allergen-specific IgG strongly attenuates the induction of allergen-induced airway inflammation and hyperresponsiveness. We further take advantage of mice with a lineage-specific deletion of FcγRIIb, in which only mast cells lack the inhibitory receptor, to establish a mechanism for this protective effect; namely, inhibitory IgG signaling in mast cells results in a suppression of type 2 inflammatory chemokines and cytokines.

## 2. Results

### 2.1. Treatment with Allergen-Specific IgG Reduces Allergen-Induced Pulmonary Macrophage and Eosinophil Infiltration

We have previously demonstrated that IgE-activated mast cells express a proinflammatory gene expression program and that allergen-specific IgG, signaling via the inhibitory Fc receptor, FcγRIIb, on mast cells suppresses not only rapid degranulation but also the production of cytokines and chemokines associated with type 2 inflammation that occurs hours after activation [21,25,26]. Given the correlation of aeroallergen-specific IgG levels both with protection from allergic symptoms in IgE-sensitized individuals and with the suppression of asthma and rhinitis in subjects receiving immunotherapy, we hypothesized that anti-inflammatory effects of IgG, exerted via FcγRIIb, might suppress an airway inflammatory response to aeroallergens in a pathway orchestrated specifically by mast cells.

To study this in vivo, we adapted a previously described a mast cell-dependent model of asthma developed by Nakae et al. that involves repeated i.p. sensitization with ovalbumin (OVA) followed by intranasal (i.n.) challenge and is characterized by airway inflammation, allergen-specific IgE production, and bronchial hyperresponsiveness [3,4]. To test the effect of allergen-specific IgG antibodies on OVA-induced airway inflammation in this model, we prepared a pool of polyclonal mouse OVA-specific IgG, prepared in OVA-immunized IgE^−/−^ mice so as to ensure the absence of any OVA-specific IgE [27]. Control IgG was prepared by pooling serum from IgE^−/−^ mice that were not immunized with OVA. To focus our analysis of IgG effects specifically to signals delivered by FcγRIIb on mast cells, we bred mice expressing Cre-recombinase under the control of the mast cell-specific Mcpt5 promoter (Mcpt5^Cre+^) with FcgRIIb^fl/fl^ mice in which the gene encoding FcγRIIb is flanked by loxP sites, the targets of the cre-recombinase. As anticipated, the Mcpt5^Cre+^FcgRIIb^fl/fl^ mice lacked FcgRIIb on their mast cells but had normal levels of the receptor on B cells and several other lineages known to physiologically express the receptor [21]. OVA (or sham)-sensitized Mcpt5^Cre+^FcgRIIb^fl/fl^ or Mcpt5^Cre+^FcgRIIb^wt/wt^ mice were subjected to OVA challenge and analyzed for airway responses as per the treatment groups outlined in Table 1.

**Figure 1:**
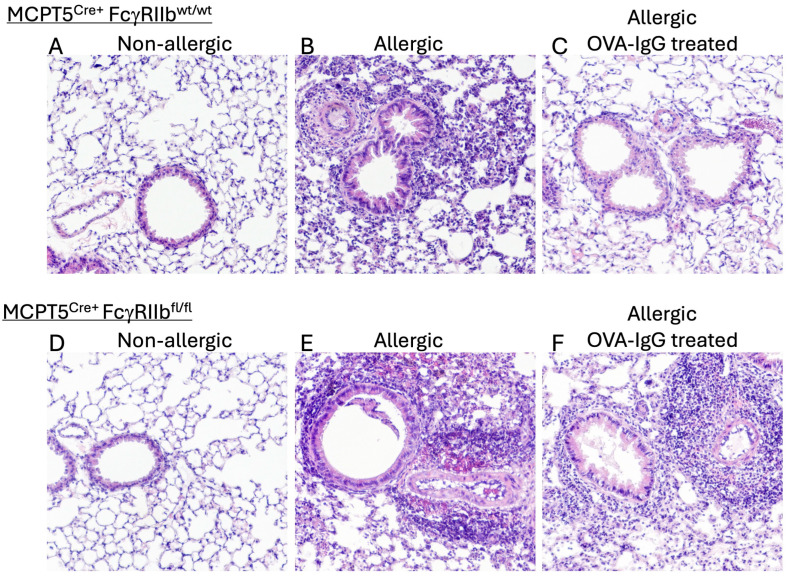
Mast cell FcγRIIb-mediated effects of IgG treatment on allergen-induced lung inflammation. Control MCPT5^Cre+^FcgRIIb^wt/wt^ mice (**A**–**C**) and MCPT5^Cre+^FcgRIIb^fl/fl^ mice (**D**–**F**) with lineage-specific deletion of FcγRIIb restricted to mast cells were subjected to OVA (or PBS control) sensitization by i.p. injection followed by repeated i.n. OVA challenge with hematoxylin and eosin–stained lung sections prepared 24 h after third i.n. OVA challenge and inspected at 100×. Representative sections are shown from among tissue samples prepared from 4 mice in each group. Experimental groups summarized in Table 1.

As expected, and previously observed by others, when compared to unsensitized controls (Figure 1A), OVA-sensitized and challenged control mice (Figure 1) exhibited a significant mixed influx of inflammatory cells surrounding the airways and blood vessels of the lungs, a response that was mirrored in Mcpt5^Cre+^FcgRIIb^fl/fl^ mice that lack FcγRIIb (Figure 1E). Treatment with 10 mg of allergen-specific IgG by i.p. injection, twice during the sensitization phase and once right before the challenge period, led to a marked reduction in inflammatory cell recruitment (1C) in Mcpt5^Cre+^FcgRIIb^wt/wt^ mice. In contrast, Mcpt5^Cre+^FcgRIIb^fl/fl^ mice, lacking FcγRIIb on their mast cells, continued to have an intense inflammatory reaction in the lungs despite IgG treatment (Figure 1F).

**Figure 2:**
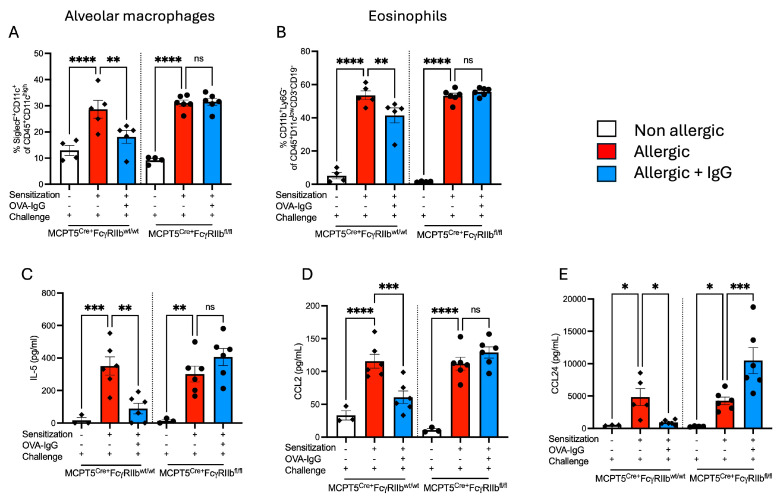
Effects of FcγRIIb-mediated IgG signaling on alveolar macrophage and eosinophil counts, and airway levels of chemoattractants. (**A**) Alveolar macrophages and (**B**) eosinophils were detected in lung single-cell suspensions by flow cytometry. (**C**–**E**) IL-5, CCL2, and CCL24 levels in allergen-sensitized and -challenged MCPT5^Cre+^FcgRIIb^wt/wt^ and MCPT5^Cre+^FcgRIIb^fl/fl^ mice with and without IgG treatment were measured in the BALF by ELISA. Groups for each strain (MCPT5^Cre+^FcgRIIb^wt/wt^ mice and MCPT5^Cre+^FcgRIIb^fl/fl^) are represented as non-allergic, allergic, and allergic + IgG. *n* = 4–6 per group per strain for (**A**,**B**), 3–6 for (**C**–**E**). * *p* < 0.05, ** *p* < 0.01, *** *p* < 0.001, **** *p* < 0.0001 and “ns” denotes non-significant comparisons. Cytokine concentrations are presented as pg/mL. Comparisons highlight immune responses across genetic backgrounds and treatment regimens.

The IgG effects observed on histopathological examination were confirmed by flow cytometry. OVA sensitization and challenge drove the robust recruitment of macrophages and eosinophils in both control Mcpt5^Cre+^FcgRIIb^wt/wt^ and Mcpt5^Cre+^FcgRIIb^fl/fl^ mice (Figure 2A,B). While the influx of both cell types was significantly reduced in IgG-treated WT mice, IgG treatment had no effect on animals lacking FcγRIIb on mast cells. The reduced inflammatory influx in animals receiving IgG suggested that the chemokines and cytokines that drive the differentiation and recruitment of these cells, which are in part produced by mast cells, might be affected by IgG treatment. Indeed, OVA-sensitized and -challenged mice displayed elevated levels of IL-5 as well as chemokine ligands 2 and 24 in the BALF (Figure 2C–E). The lungs of control Mcpt5^Cre+^FcgRIIb^wt/wt^ but not Mcpt5^Cre+^FcgRIIb^fl/fl^ animals treated with allergen-specific IgG exhibited a marked attenuation in the induction of these mediators and this suppression was almost entirely dependent upon the expression of FcγRIIb on mast cells (Figure 2).

### 2.2. Goblet Cell Hyperplasia Induced Following Allergen Sensitization and Challenge Is Reduced by Allergen-Specific IgGF

**Figure 3:**
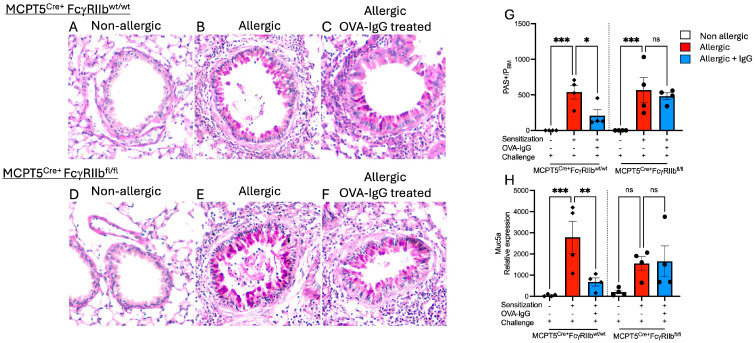
Effects of IgG treatment on goblet cell metaplasia in OVA-sensitized and -challenged mice. (**A**–**F**) Periodic Acid–Schiff (PAS)-stained lung sections showing goblet cells in bright pink. (**G**) Goblet cell hyperplasia was determined at 100× by counting the number of PAS+ cells per airway and standardized for airway size by dividing by the perimeter of the basement membrane (P_BM_). (**H**) Relative expression levels of Muc5a, a key mucin gene as determined by RT-qPCR. *n* = 4–5 per group per strain; sections are representative selections from among 4 tissue samples prepared for each group. * *p* < 0.05, ** *p* < 0.01, *** *p* < 0.001, and “ns” denotes non-significant difference.

In addition to inducing the influx of inflammatory cells, allergen inhalation is known to drive goblet cell metaplasia in the airway epithelium. Mucus production by these goblet cells is responsible in part for the and airway obstruction characteristic of asthma. To determine if allergen-specific IgG, in addition to blocking airway inflammation, might inhibit the expansion of goblet cells, we performed periodic acid–Schiff (PAS) staining on lung sections. Non-allergic Mcpt5^Cre+^ controls and mast cell FcγRIIb-deficient Mcpt5^Cre+^FcgRIIb^fl/fl^ mice did not have PAS-positive cells in their airways (Figure 3A,D,G), whereas allergic mice (OVA-sensitized and -challenged) mounted a substantial increase in PAS-positive cells (Figure 3B,E,G). In mice treated with allergen-specific IgG, this increase in PAS-positive cells was blunted but only in animals expressing FcγRIIb on mast cells (Figure 3B vs. Figure 3C and Figure 3F vs. Figure 3G).

Consistent with the increase in mucin-producing cells seen on tissue sections, expression of the mucin-associated gene Muc5ac was elevated in OVA-sensitized and -challenged mice (Figure 3H). Allergen-exposed mice treated with specific IgG exhibited reduced Muc5ac expression, correlating with the observed decrease in PAS staining. Similarly to the findings for goblet cell hyperplasia, this reduction was dependent upon FcγRIIb expression on mast cells.

### 2.3. Allergen-Specific IgG Inhibits the Production of Type 2 Cytokines and IgE

**Figure 4:**
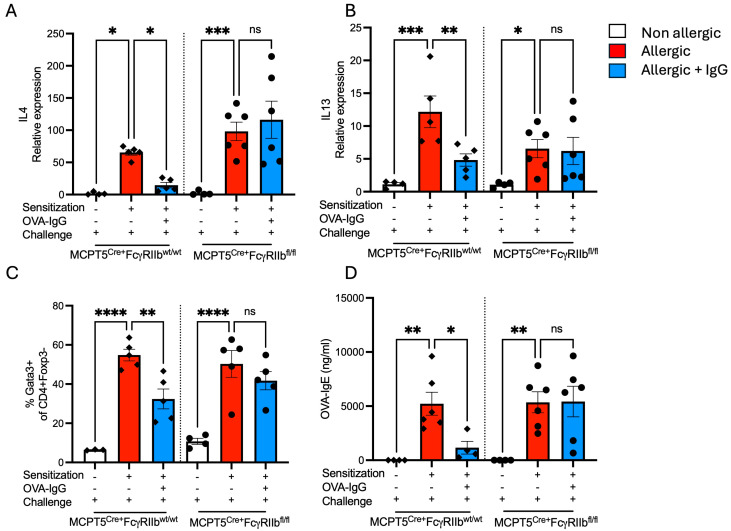
Th2 cytokine gene expression and T-cell phenotype. Relative expression of (**A**) IL-4 and (**B**) IL-13 transcripts in lung tissue. (**C**) Percentage of Gata3^+^ CD4^+^ Foxp3^−^ T cells, representing Th2 cells. (**D**) Serum levels of OVA-specific IgE. *n* = 4–6 per group per strain. * *p* < 0.05, ** *p* < 0.01, *** *p* < 0.001, **** *p* < 0.0001 and “ns” denotes non-significant differences. The experimental setup includes sensitization, OVA-IgG treatment, and allergen challenge to evaluate genotype-specific immune responses in control MCPT5^Cre+^FcgRIIb^wt/wt^ mice and MCPT5^Cre+^FcgRIIb^fl/fl^ mice.

In addition to inducing the influx of inflammatory cells, allergen inhalation is known to drive Allergic asthma, type 2 tissue inflammation and goblet cell expansion are driven by Th2 cytokines. The lungs of OVA-sensitized and -challenged mice had a robust induction of IL-4 and IL-13 transcripts that was suppressed by IgG only in mice with FcγRIIb sufficient mast cells (Figure 4A,B). In parallel, the frequency of Th2 cells, as characterized by Gata3 expression, was increased following OVA sensitization and challenge that was attenuated by IgG treatment in an FcγRIIb-dependent fashion (Figure 4C). The production of allergen-specific IgE antibodies, the hallmark of allergic diseases, is completely dependent on the Th2 cytokine, IL-4. Consistent with the pattern observed for Th2 cytokines, sensitized and challenged mice mounted strong IgE responses which were suppressed by IgG only in mice with normal FcγRIIb expression (Figure 4D). Notably, the ability of Mcpt5^Cre+^FcgRIIb^fl/fl^ mice to generate robust OVA-IgE titers even following IgG treatment confirms that IgG in this model does not inhibit antibody responses per se but rather exerts its effects via FcγRIIb by regulating Th2 tone and IgE isotype switching.

### 2.4. Expansion of Mucosal Mast Cells in the Allergic Lung Is Attenuated by Allergen-Specific IgG Treatment

**Figure 5:**
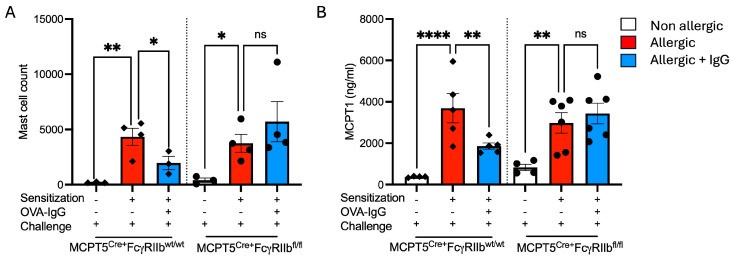
Mast cell percentages and mast cell protease-1 (MCPT1) levels in allergen + IgG-treated mice. (**A**) Mast cell percentages in digested lung by flow cytometry across non-allergic, allergic, and allergic + OVA-IgG-treated groups in control MCPT5^Cre+^FcgRIIb^wt/wt^ mice and MCPT5^Cre+^FcgRIIb^fl/fl^ mice. (**B**) Levels of serum MCPT1 (ng/mL) following sensitization and allergen challenge with and without IgG treatment. *n* = 3–4 per group per strain for (**A**), 4–6 for (**B**). * *p* < 0.05, ** *p* < 0.01, **** *p* < 0.0001, and “ns” denotes non-significant differences.

As mast cells are widely recognized as central effector cells both of acute responses to aeroallergen inhalation as well as key inducers of chronic allergic inflammation and have been shown to be expanded in the airways of subjects with severe asthma, we wanted to investigate the effects of allergen-specific IgG on lung mast cell numbers. OVA-sensitized and -challenged, sham IgG-treated mice exhibited a marked mast cell expansion in the lungs compared to non-allergic controls. Treatment with ovalbumin-specific IgG markedly blunted this increase (Figure 5A) and this suppression was dependent upon FcγRIIb expression on mast cells. Serum MCPT1, a mast cell granule protease the presence of which in plasma reflects recent mast cell activation, was undetectable in non-allergic mice (Figure 5B). However, following challenge of sensitized animals, serum MCPT1 levels were increased. Allergen-specific IgG treatment decreased circulating MCPT1 levels, dependent upon FcγRIIb expression. Mice lacking FcγRIIb on mast cells retained elevated circulating MCPT1 levels (Figure 5B).

### 2.5. Increases in Airway Responsiveness Following Allergen Sensitization and Challenge Are Reduced with Allergen-Specific IgG Treatment

**Figure 6:**
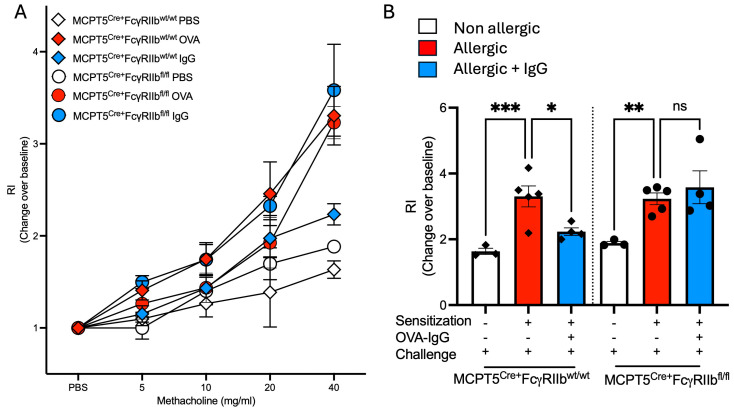
Airway responsiveness in MCPT5^Cre+^ FcgRIIb^wt/wt^ and MCPT5^Cre+^ FcgRIIb^fl/fl^ mice following sensitization and allergen challenge with and without OVA-IgG treatment. (**A**) Dose–response curve showing airway responsiveness to increasing concentrations of methacholine (5–40 mg/mL) in non-allergic, allergic, and allergic + OVA-IgG-treated groups in control MCPT5^Cre+^FcgRIIb^wt/wt^ mice and MCPT5^Cre+^FcgRIIb^fl/fl^ mice. (**B**) Respiratory index (RI) at 40 mg/mL of methacholine measured the change from baseline across different treatment conditions as relative (non-allergic, allergic, and allergic + OVA-IgG) in both strains. *n* = 3–5 per group per strain. * *p* < 0.05, ** *p* < 0.01, *** *p* < 0.001, and “ns” denotes non-significant differences.

The protective effects of allergen-specific IgG in normalizing levels of key allergic response mediators and airway inflammation prompted us to assess its impact on allergen-induced changes in lung physiology. Airway responsiveness (AHR) to methacholine was measured by methacholine challenge in which OVA-sensitized and challenged mice from both Mcpt5^Cre+^FcgRIIb^wt/wt^ Mcpt5^Cre+^FcgRIIb^fl/fl^ strains exhibited significant shifts in methacholine dose responsiveness (Figure 6A). As expected, given the patterns of airway inflammation, goblet cell metaplasia and cytokine expression we had observed in these animals, Mcpt5^Cre+^FcgRIIb^wt/wt^, but not Mcpt5^Cre+^FcgRIIb^fl/fl^ mice had a significant blunting of allergen-induced AHR as is most evident at the 40 mg/mL dose of methacholine (Figure 6B).

## 3. Discussion

The presence of allergen-specific IgG antibodies is known to correlate with protection from symptoms in subjects harboring aeroallergen-specific IgE and the induction of such antibodies during immunotherapy tracks with clinical improvement. These observations point to an inhibitory role for IgG in allergic pathogenesis but the mechanistic basis for IgG-mediated protection has not been fully explored. Our previous studies in food allergy, using clinical samples as well as murine models, have shown that IgG, in an FcγRIIb-dependent manner, can suppress both immediate mast cell and basophil activation by IgE:allergen and the induction of systemic Th2-adaptive immune responses by mast cell-derived IL-4 in allergen-exposed mice [25,26,28]. Recently, using a panel of allergen-specific IgE and IgG antibodies along with cultured mast cells from FcγRIIb-sufficient and -deficient mice, we found that allergen:IgE-mediated mast cell activation induces a strong type 2 proinflammatory transcriptional program and that IgG, in an FcγRIIb-dependent manner, blocks the induction of chemokine and cytokine transcripts and proteins and inhibits inflammatory cell recruitment to the peritoneum of mice given intraperitoneal allergen challenges [21].

Taken together, these observations suggest that IgG via FcγRIIb might attenuate inflammatory tissue responses in allergic disease. Here we provide strong evidence in support of that hypothesis in a mouse model of asthma, showing that IgG antibodies suppress recruitment of inflammatory cells to the airway as well as the production of IgE antibodies and the cytokines and chemokines that drive this response, attenuating goblet cell metaplasia and mast cell expansion, and blocking the induction of AHR. Notably, these IgG effects were absent in mice lacking FcγRIIb specifically on mast cells, implicating suppression of mast cell activation as an essential component of IgG-mediated protection.

FcγRIIb is expressed on many cell types and its critical function in regulating antibody responses via negative signaling in B cells has been extensively studied [29,30]. However, the finding that FcγRIIb signals restricted to mast cells can exert broad immunoregulatory effects on tissue inflammation is novel and implicates mast cells as a central nidus in both the induction and suppression of allergic disease. Previously, adjuvant free asthma models, in which the proinflammatory effects of mast cells become most apparent because of the elimination of adjuvant-driven inflammatory processes, have been used by Galli and others to define a key endogenous adjuvant function for mast cells themselves, perhaps driven in part by their production of TNF, in orchestrating allergen driven type 2 lung inflammation [31]. For instance, Nakae et al., using the same adjuvant-free model we employed in the current study, found that the *Kit^Wsh/Wsh^* mast cell-deficient mice exhibited markedly reduced eosinophil recruitment into the airways, lung inflammation, pulmonary Th2 cytokine production, and airway hyperreactivity [3]. The reconstitution of these mice with wild-type mast cells restored the deficient responses, confirming that mast cells themselves, can drive these asthma-associated phenotypes [3]. Such observations have given rise to the concept of mast cells as “tunable” effector and immunoregulatory cells [32]. Our own studies, mostly in models of food allergy and using an inducible mast cell deficient line of mice, *Mcpt5^cre^ iDTR*, have further implicated IgE-activated mast cells in the regulation of Th2 responses and IgE antibody production along with suppression of regulatory T cells [28]. We observed that these pro-Th2 and anti-regulatory T-cell functions of IgE-activated mast cells were suppressed by IgG in an FcγRIIb-dependent manner [25,26]. While these previous findings established a central role for regulating adaptive immune responses to allergens, the food allergy disease process is not associated with a significant inflammatory component. Our current study allowed us to extend the analysis to allergic airway phenotypes and, most importantly to airway inflammation. Consistent with the prior studies, mast cells were again implicated as disease drivers and IgG:FcγRIIb as suppressors of their proinflammatory functions.

In an interesting observation regarding the yin and yang of IgE and IgG antibodies in allergy, our results additionally implicate these opposing signals in the regulation of tissue mast cell homeostasis. A marked increase in mast cell numbers was elicited in the airways of OVA-exposed mice, a response previously observed by others [2,33]. Brightling and colleagues have similarly shown that mast cell expansion occurs in subjects with asthma [1]. OVA-induced mast cell expansion was inhibited by IgG antibodies signaling via FcγRIIb. Roles for allergen exposure and IgE:FcεRI and IgG:FcγRIIb signals in mast cell homeostasis have previously been demonstrated in food allergy models [25,26,34,35]. Taken together these observations point to the IgG:FcγRIIb axis as an important physiologic checkpoint in mast cell homeostasis that could be considered a therapeutic target in mast cell-mediated diseases.

The appearance of monocytes and eosinophils in the lungs of OVA-treated mice along with the IgG:FcγRIIb-mediated suppression of this recruitment prompted us to look at levels of IL-5, CCL2, and CCL24, known drivers of this type of influx, within the airways. As has been observed by others in murine asthma models, we could easily detect IL-5 in the BALF of OVA-sensitized and -challenged animals. Of course, IL-5 is well known for its role in eosinophilopoiesis but it also has important functions in eosinophil recruitment, acting directly as a chemoattractant and also priming eosinophils for chemokine-mediated chemotaxis [36]. CCL2, which we detected in BAL, is a chemoattractant for monocytes and basophils while CCL24, also known as eotaxin-2, recruits eosinophils and basophils, is elevated in some forms of asthma and functions to induce inflammation after bronchial allergen challenge [37,38,39]. The decrease in each of these mediators following allergen-specific IgG treatment further supports the conclusion that IgG can downregulate proinflammatory pathways in allergic responses.

The presence of OVA-induced goblet cell hyperplasia is typical in murine asthma models and is a key feature of chronic allergic inflammation. As expected, the significant increases in PAS-positive goblet cells we observed in allergic mice in the current study correlated with increased pulmonary IL-13, a key driver of goblet cell metaplasia, and *Muc5ac* expression, a marker of goblet cell function. Along with elevated IL-13 transcripts in the lung, we observed IgG-suppressible induction of IL-4 and increases in Gata3^+^ T cells in OVA-treated mice, both indicators of a local Th2 dominant response to allergen, a response that underlies the airway hyperresponsiveness that is the physiologic hallmark of asthma.

Collectively, our results support multifaceted effects of allergen-specific IgG in regulating mast cell-driven allergic airway inflammation, reducing cellular infiltration, suppressing goblet cell metaplasia, influencing mast cell function and homeostasis, and mitigating airway obstruction and AHR. The dependence on mast cell FcγRIIb expression highlights a specific therapeutic target that could be leveraged in the development of new treatments for allergic diseases. Indeed, aeroallergen IgG monoclonal antibody cocktails have shown promise in the treatment of allergy with inhibition of mast cell and basophil activation in vivo, reductions in nasal Th2 cytokine and chemokine levels and protection from airflow obstruction (drop in FEV1) and reduced nasal symptom scores following allergen challenge in cat- and birch pollen-sensitive subjects [18,19,40,41]. Future studies will hopefully integrate mechanistic analyses of the beneficial effects of IgG in such trials, specifically focusing on the role of FcγRIIb so that more refined versions of such therapies can be developed.

## 4. Materials and Methods

### 4.1. Mice

All animal studies were conducted under protocols approved by the Boston Children’s Hospital Institutional Animal Care and Use Committee. Mice were bred and maintained in specific pathogen-free conditions, housed in individually ventilated cages with a maximum of five adults per cage. All mice were of the C57BL/6 background. MCPT5^Cre+^ and FcgRIIb^fl/fl^ mice were generously provided by Drs. Axel Roers and Sjef Verbeek, respectively [42,43]. MCPT5^Cre+^FcgRIIb^fl/fl^ mice were generated by crossing MCPT5^Cre+^ mice with FcgRIIb^fl/fl^ mice.

### 4.2. Allergic Asthma Protocol

An adjuvant-free allergic airway inflammation protocol was adapted from Nakae et al. [3]. Mice were sensitized via intraperitoneal (i.p.) injection of 10 µg of ovalbumin (100 µL of a 100 µg/mL solution prepared in PBS) every other day for a total of seven injections. Three weeks after the last injection, mice were intranasally challenged with 50 µg of ovalbumin (25 µg per nostril from a 1 mg/mL solution) every third day for a total of three challenges. 24 h after the final challenge, animals were euthanized, and samples were collected. Mice were treated with 10 mg of OVA-IgG or control/sham IgG 24 h prior to the first and last sensitizations and 24 h prior to the last challenge. For each strain, the unsensitized group received i.p. PBS injections during the sensitization period, i.p. sham IgG treatment, and i.n. ovalbumin challenge.

### 4.3. Bronchoalveolar Lavage Fluid (BALF) Collection

Following euthanasia, mice underwent tracheostomy, and a flexible cannula was inserted to wash the lungs three times with 1 mL of PBS to collect the bronchoalveolar lavage (BAL). Cells in the BAL were separated from the fluid by centrifugation (1200 RPM for 5 min, and the lavage fluid was stored with a protease inhibitor cocktail at −80 °C until further analysis.

### 4.4. CCL2, CCL24 and IL-5 ELISA

Mouse CCL2, CCL24, and IL-5 ELISAs were performed using commercially available ELISA kits following manufacturer’s instructions (R&D Systems, Minneapolis, MN, USA).

### 4.5. Lung Immune Cell Phenotyping

Lung digestion was performed as previously described [33]. Briefly, two lung lobes were minced and incubated for 45 min at 37 °C with shaking (220 RPM) in 5% FBS/PBA containing 2.2 mg/mL of collagenase type 4 and 100 µg/mL of DNase using gentleMACS C tubes (Miltenyi Biotec, Auburn, CA, USA). Further digestion was carried out using the LUNG-02 protocol on a gentleMACS tissue dissociator (Miltenyi Biotec, Auburn, CA, USA). After digestion, the sample was centrifuged at 1400 RPM, and red blood cell lysis was performed. The resulting sample was passed through a 70 µm strainer to obtain a single-cell suspension.

To identify mast cells, lung cells were stained for viability (APC-Cy7), CD45 (AF700, clone 30-F11), and lineage markers for negative selection (all in FITC: CD4 clone GK1.5, CD8a clone 53-6.7, CD19 clone 6D5, CD11c clone M1/70, CD11b clone N418), along with cKIT (BV650, clone 2B8), FcεRIa (PE-Cy7, clone MAR-1), and IgE (PE, clone RME-1) (Biolegend, San Diego, CA, USA).

Eosinophils were identified as CD45^+^ CD11c^low^, CD3^−^, CD19^−^, Ly6G^−^, and CD11b^+^. Alveolar macrophages were identified as CD45^+^, CD11c^high^, CD11b^+^, and Ly6G^+^.

### 4.6. MCPT1 ELISA

To detect serum levels of MCPT1, blood was collected from mice two hours after the third intranasal antigen challenge. MCPT1 levels were measured using an MCPT-1 ELISA Kit (Thermo Fisher Scientific, Carlsbad, CA, USA) according to the manufacturer’s instructions.

### 4.7. OVA-IgE ELISA

OVA-specific IgE levels were assessed via sandwich ELISA using serum collected from mice at the experimental endpoint. ELISA plates were coated with 1 µg/mL goat anti-mouse IgE (Southern Biotech, Birmingham, AL, USA) overnight at 4 °C. The following day, plates were blocked with 2% BSA-PBS for two hours at room temperature. Sera were added at various dilutions and incubated overnight at 4 °C to capture IgE antibodies. After washing with 1X PBS containing 0.028% Tween, biotinylated OVA diluted in 2% BSA-PBS was added and incubated for one hour at room temperature. Plates were washed again and incubated with streptavidin-horseradish peroxidase for 30 min at room temperature. The ELISAs were developed using 3,3′,5,5′-Tetramethylbenzidine (TMB; Thermo Fisher Scientific) and the reaction was stopped with 2 N H_2_SO_4_. Plates were read at 450 nm.

### 4.8. RNA Extraction, cDNA Synthesis, and RT-qPCR

Lung sections were collected from mice and stored in RNAlater (Thermo Fisher, Carlsbad, CA, USA) at 4 °C overnight before being frozen at −80 °C. RNA was extracted from 20 to 30 mg of tissue using the Qiagen RNeasy Mini Kit (Qiagen, Hilden, Germany) according to the manufacturer’s instructions. RNA concentrations were measured using a Nanodrop 2000 (Thermo Fisher, Carlsbad, CA, USA). One microgram of RNA was reverse transcribed into cDNA using the iScript cDNA Synthesis Kit (Bio-Rad, Hercules, CA, USA) following the manufacturer’s protocol. Gene expression was quantified using the QuantStudio 3 real-time qPCR instrument (Thermo Fisher, Carlsbad, CA, USA), TaqMan Fast Advanced Master Mix (Applied Biosystems, Thermo Fisher, Carlsbad, CA, USA), and pre-designed probes (Life Technologies, Thermo Fisher, Carlsbad, CA, USA). Gene expression was normalized to Gapdh and calculated using the Cq values and the 2^−ΔΔCq^ method.

### 4.9. Generation of Ovalbumin-Specific IgG Antibodies

IgE knockout mice (Igh-7 [27]) were immunized with ovalbumin via i.p. injection once a week for three consecutive weeks, using 100 µg of ovalbumin (OVA; Sigma-Aldrich, St. Louis, MO, USA) adsorbed to 1.5 mg of aluminum hydroxide (Imject Alum, Pierce, Rockford, IL, USA) in 0.2 mL of sterile PBS. One week after the final sensitization, mice were euthanized, and blood was collected via cardiac puncture. Serum was pooled from multiple mice. IgG was isolated using protein G spin columns (Thermo Scientific, Waltham, MA, USA), concentrated, and dialyzed with Amicon Ultra centrifugation devices, then sterile filtered through 0.2 µm syringe filters. OVA-specific IgG1 concentrations were determined by ELISA, and mice were injected with 10 µg of IgG1. Control IgG was prepared from mock-immunized mice.

### 4.10. Histology

Lungs were removed from the mice, inflated with 10% buffered formalin, and immersed therein for a minimum of 36 h. The lungs were then trimmed, dehydrated, embedded in paraffin, and cut into 3 μm thick sections. The sections were deparaffinized, hydrated and stained with the hematoxylin and eosin (H&E) and periodic acid Schiff (PAS) stains to quantify inflammation, and goblet cell hyperplasia, respectively. Dehydration, paraffin embedding, sectioning, and staining were completed by the Harvard Medical Area Rodent Histopathology Core (Harvard, Boston, MA, USA). Images were captured on a Nikon E800 microscope (Nikon, Melville, NY, USA).

### 4.11. Measurement of Goblet Cell Hyperplasia

PAS-stained slides were used to quantify goblet cell hyperplasia in the lungs. PAS-positive cells within the airway were counted. Data was normalized by dividing the counts by the perimeter of the basement membrane (P_BM_). ImageJ (version 1.54j) was used to count the cells and measure the P_BM_. Per mouse, at least four airway cross-sections coming from the same depth were counted.

### 4.12. Measurement of Airway Hyperresponsiveness

At 24 h after the third allergen challenge, mice were anesthetized then tracheotomized and connected to a ventilator. A nebulizer was used to administer ascending doses of methacholine (PBS, 5, 10, 20, and 40 mg/mL). The maximum responsiveness value for each mouse at each dose of methacholine was determined using a Buxco plethysmograph system and Harvard Apparatus ventilators (Harvard Apparatus, Holliston, MA, USA).

### 4.13. Statistical Analysis

The results are presented as mean ± SEM. Ordinary one-way ANOVA with multiple comparisons was performed using Prism version 10.3.1 (GraphPad Software, San Diego, CA, USA). *p* values are indicated in the figures using the shorthand * *p* < 0.05, ** *p* < 0.01, *** *p* < 0.001, and **** *p* < 0.0001.

## Figures and Tables

**Table 1:** Mouse strains, FcγRIIb status, IgG treatment groups, and ovalbumin challenge groups.

Genotype	FcγRIIb	Sensitization Allergen	OVA-IgG	Challenge Allergen
MCPT5^Cre+^ FcgRIIb^wt/wt^	Present	PBS	−	OVA
		OVA	−	OVA
		OVA	+	OVA
MCPT5^Cre+^ FcgRIIb^fl/fl^	Absent	PBS	−	OVA
		OVA	−	OVA
		OVA	+	OVA

## Data Availability

The original contributions presented in this study are included in the article. Further inquiries can be directed to the corresponding author.

## Abbreviations

The following abbreviations are used in this manuscript:

AHR	airway hyperresponsiveness
BALF	bronchoalveolar lavage fluid
CCL	chemokine ligand
ELISA	enzyme-linked immunosorbent assay
Fl	flox
H and E	hematoxylin and eosin
Ig	immunoglobulin
IL	interleukin
i.n.	intranasal
i.p.	intraperitoneal
MCPT1	mast cell protease 1
Muc5a	mucin 5a
OVA	Ovalbumin
PAS	periodic acid-Schiff
wt	wildtype
